# Comparing Intramedullary Nails and Locking Plates in Displaced Proximal Humerus Fracture Management: A Systematic Review and Meta-Analysis

**DOI:** 10.7759/cureus.54235

**Published:** 2024-02-15

**Authors:** Stacey S D'Almeida, Reily Cannon, Nguyen T Vu, Brent A Ponce, David Redden

**Affiliations:** 1 General Medicine, Oceania University of Medicine, Auckland, NZL; 2 Medicine, Touro College of Osteopathic Medicine, Henderson, USA; 3 Medicine, Edward Via College of Osteopathic Medicine, Auburn, USA; 4 Orthopedic Surgery, Hughston Clinic, Columbus, USA; 5 Research and Biostats, Edward Via College of Osteopathic Medicine, Auburn, USA

**Keywords:** range of motion (rom), functional scores, locking plate, intramedullary nailing (imn), proximal humeral fracture

## Abstract

This study aims to provide an updated review comparing the complication rates and clinical outcomes of intramedullary nails and locking plates (LPs) in displaced proximal humerus fracture (PHF) management. We performed a systematic review of the Cochrane Central Register of Controlled Trials, Clinical Trials Registry, EMBASE, and PubMed. Studies with level III evidence or higher comparing intramedullary nails and LPs used for internal fixation of displaced PHFs were included. The Methodological Index for Nonrandomized Studies (MINORS) criteria and Cochrane Handbook for Systematic Reviews of Interventions 5.2.0 were used to assess the risk of bias. Our meta-analysis included a comparison of method-related complications, pain scores, range of motion (ROM), and functional scores. A total of 13 comparative studies were included: five randomized controlled trials, three prospective cohort studies, and five retrospective cohort studies. The total number of patients included was 1,253 (677 in the LP group and 576 in the intramedullary nail group). Superior Constant-Murley scores and external rotation ROM were found in the LP group during the early postoperative period. However, long-term functional scores and complication rates were comparable between the two groups. We conclude that intramedullary nailing and LP fixation are both equally effective for the treatment of displaced PHFs. Neither treatment appears superior at this time, and more large-scale randomized controlled trials should be conducted to further evaluate the potential benefit of LPs in the early postoperative period.

## Introduction and background

Proximal humerus fractures (PHFs) are common, accounting for 5%-6% of all adult fractures [1]. While approximately 58% of PHFs are considered displaced, only 21% are treated surgically due to factors such as the patient’s condition, bone quality, surgeon experience, and comparable outcomes with non-operative management [2-4]. Iglesias-Rodríguez et al. demonstrated a comparable incidence of fractures in both males and females at younger ages. This is contrasted with an increase in female PHFs beginning at age 50 [5]. PHFs typically arise in two distinct populations: high-energy injuries, more common in males, and low-energy injuries, predominately in females [6]. The most recent Cochrane review on PHF management demonstrated insufficient evidence for proper management of high-energy fractures and for those under age 60; comparable outcomes were found in surgical vs. nonsurgical treatment of low-energy fractures [3]. The lack of a definitive treatment direction for displaced PHFs stems from two distinct issues: the paucity of literature regarding high-energy injuries and ambiguity regarding a gold standard treatment for low-energy fractures.

PHFs in the elderly population often meet the criteria for osteoporotic fractures [7,8] and comprise the third most common osteoporotic fracture type [9]. Osteoporotic fractures make up 30% of fractures in men, 66% of fractures in women, and 70% of inpatient fractures [10]. Arthroplasty and non-operative measures are often considered for fragility fractures, as individuals with these fractures often have comorbidities that need to be evaluated in determining optimal management. A 2022 investigation identified a one-year mortality rate of 17.4% following a fragility PHF and a 15.3% rate of rehospitalization for medical issues in the immediate post-injury period [11]. This concern is further validated by an insufficient amount of evidence regarding an evidence-based gold standard treatment for displaced PHFs [3,12]. The prevalence of PHFs combined with the aging population and the morbidity burden of these fractures calls for frequent evidence-based assessments of fixation options.

Intramedullary (IM) nailing and locking plate (LP) fixation are two surgical options intended to restore function in patients with displaced PHF [13]. While open reduction and internal fixation (ORIF) with LP fixation is the most common surgical intervention, this intervention is associated with a high risk of complications [14]. A 2011 systematic review of humeral LP complications indicated a 19.5% complication rate for intraarticular screw penetration, 6.8% for varus collapse, 5.0% for subacromial impingement, 4.6% for avascular necrosis, 4% for adhesive capsulitis, 1.5% for nonunion, 1.4% for deep infection, and 13.8% reoperation rate [15]. Zhu et al. identified a 31% complication rate in the LP group and a 4% rate in the locking IM nail group. At three-year follow-up, both surgical methods obtained similar results [16]. The most recent meta-analysis in 2019 showed superior results with IM nailing regarding intraoperative blood loss, operative time, fracture healing time, postoperative complications, and postoperative infections [17]. These results indicate the possibility of an improved outcome of IM nails in comparison to LPs but are limited by the quality of evidence.

We conducted a systematic review and meta-analysis of all randomized clinical trials, prospective comparative, and retrospective comparative studies comparing the treatment of displaced PHFs with LP fixation and IM nailing, measured in terms of clinical outcomes (functional scores and range of motion [ROM]) and complications.

## Review

Methodology

Eligibility Criteria

The 2020 Preferred Reporting Items for Systematic Reviews and Meta-analyses (PRISMA) guidelines and the PICOS (Population, Intervention, Control, Outcome, and Study Design) framework were used to guide the search process [18,19]. Randomized clinical trials and comparative studies of level III evidence or higher with a minimum of six months of follow-up were eligible. There were no restrictions in language or years of publication. Unpublished manuscripts and conference abstracts were not included.

Using the PICO framework, a comprehensive search was conducted for all papers concerning the internal reduction of displaced PHFs with a minimum of two treatment groups. All papers must have included both LPs and intramedullary nails, and measure clinical outcomes and/or method-related complications.

Information Sources

PubMed, EMBASE, Clinical Trials Registry, and Cochrane Central Register of Controlled Trials were used in the database search, each last accessed on August 3, 2022. References cited in study reports included in the systematic review and previous systematic reviews were examined, and additional potential studies were identified with Google Scholar (https://scholar.google.com/).

Search Strategy

We used the following medical subject headings (MeSH) and keywords to search PubMed, EMBASE, and Cochrane Central Register of Controlled Trials: (shoulder fractures OR proximal humeral fracture OR proximal humeral fractures OR proximal humerus fracture OR proximal humerus fractures OR proximal humerus OR humerus surgical neck fracture OR humerus surgical neck fractures OR humeral surgical neck fracture OR humeral surgical neck fractures) AND (bone nails OR fracture fixation, intramedullary OR intramedullary nail OR intramedullary nails OR intramedullary nailing OR nail OR nails OR nailing OR IMN OR IN) AND (bone plates OR locking plate OR locking plates OR locking plating OR plate OR plates OR plating OR plate synthesis OR plating synthesis).

Data from the Clinical Trials Registry was obtained through four separate searches. Search 1: "Condition or Disease: Proximal Humeral Fracture, Study Type: All Studies, Study Results: All Studies, Intervention/Treatment: bone nails OR fracture fixation, intramedullary OR intramedullary nail OR intramedullary nails OR intramedullary nailing OR nail OR nails OR nailing OR IMN OR IN." Search 2: "Condition or Disease: Humeral Fracture, Proximal, Study Type: All Studies, Study Results: All Studies, Intervention/Treatment: bone nails OR fracture fixation, intramedullary OR intramedullary nail OR intramedullary nails OR intramedullary nailing OR nail OR nails OR nailing OR IMN OR IN." Search 3: "Condition or Disease: Humeral Fracture, Proximal, Study Type: All Studies, Study Results: All Studies, Intervention/Treatment: bone plates OR locking plate OR locking plates OR locking plating OR plate OR plates OR plating OR plate synthesis OR plating synthesis." Search 4: "Condition or Disease: Proximal Humeral Fracture, Study Type: All Studies, Study Results: All Studies, Intervention/Treatment: bone plates OR locking plate OR locking plates OR locking plating OR plate OR plates OR plating OR plate synthesis OR plating synthesis." No date or language limits were applied during the search process.

Selection Process

Two researchers (RC and SD) individually screened the title and abstract of each record. Disagreements were resolved with discussion. Articles not in the English language were excluded. The only automation tool used in the study selection process was the application of the *Randomized Controlled Trial* (RCT) and *Comparative Study* filter on PubMed. This refined 1,127 of the 1,430 PubMed results.

Data Collection Process

Two reviewers (RC and DR) manually and individually collected data from each report. Disagreements were resolved by discussion.

Three outcome domains were identified: complications, functional outcomes, and patient-centered outcomes. The complications were measured by incidence of complication and stratified by type. Functional outcomes were measured by the ROM: flexion, external rotation, internal rotation, and abduction. Patient-centered outcomes were assessed using the Visual Analog Scale (VAS), Disabilities of the Arm, Shoulder, and Hand (DASH) score, and American Shoulder and Elbow Surgeons (ASES) score. All data were measured either at the end of the follow-up period or at designated time points. The measurement time points for each included study were specified in the results. There was a minimum of six months for the follow-up period. All results that were compatible with each outcome domain in each study were sought.

*Risk-of-Bias Assessment of the *Study

Two reviewers (NV and RC) assessed the risk of bias for all the included studies. The risk of bias for included randomized clinical trials was assessed using the Cochrane Handbook for Systematic Reviews of Interventions 5.2.0 [20]. The risk of bias in the comparative studies was assessed using the Methodological Index for Nonrandomized Studies (MINORS) criteria [21]. The MINORS inclusion criteria were set to >15. Disagreements were resolved by discussion.

Eligibility for Synthesis

Study eligibility was determined by manually reading the manuscript and filtering using the following inclusion and exclusion criteria. The inclusion criteria were as follows: (1) comparative studies with level III evidence or higher; (2) internal fixation of displaced PHFs; (3) inclusion of both locking plates (LPs) and intramedullary nails; (4) a minimum of 6 months of follow-up; (5) a minimum of 8 patients for a given study; (6) clinical outcomes during follow-ups included at least one of the following: functional scores, ROM, or method-related complications. Exclusion criteria were as follows: (1) unpublished data or repeated data; (2) abstracts, letters, or proceedings of meetings; (3) cadaver model or animal experiments; (4) patients with pathologically, metabolically induced, or open fractures; (5) nondisplaced PHFs.

Statistical Synthesis Methods

Meta-analysis was conducted using both fixed and random-effect models. The statistical analysis was performed using R 4.02 statistical software with the package Meta Version 6.1-0. The analysis was completed by one reviewer (DR).

Effect Measures

The mean difference (MD) with a fixed-effect model and 95% confidence intervals (CIs) were used for continuous variables. Categorical data were analyzed using fixed- and random-effect models, odds ratios, and 95% CIs.

Certainty Assessment

CIs for random effect estimates are based on standard normal distributions. CIs for individual studies for MDs are based upon the t-distribution. CIs for individual studies for odds ratios are based on the standard normal distribution.

Results

Study Characteristics

**Figure 1:**
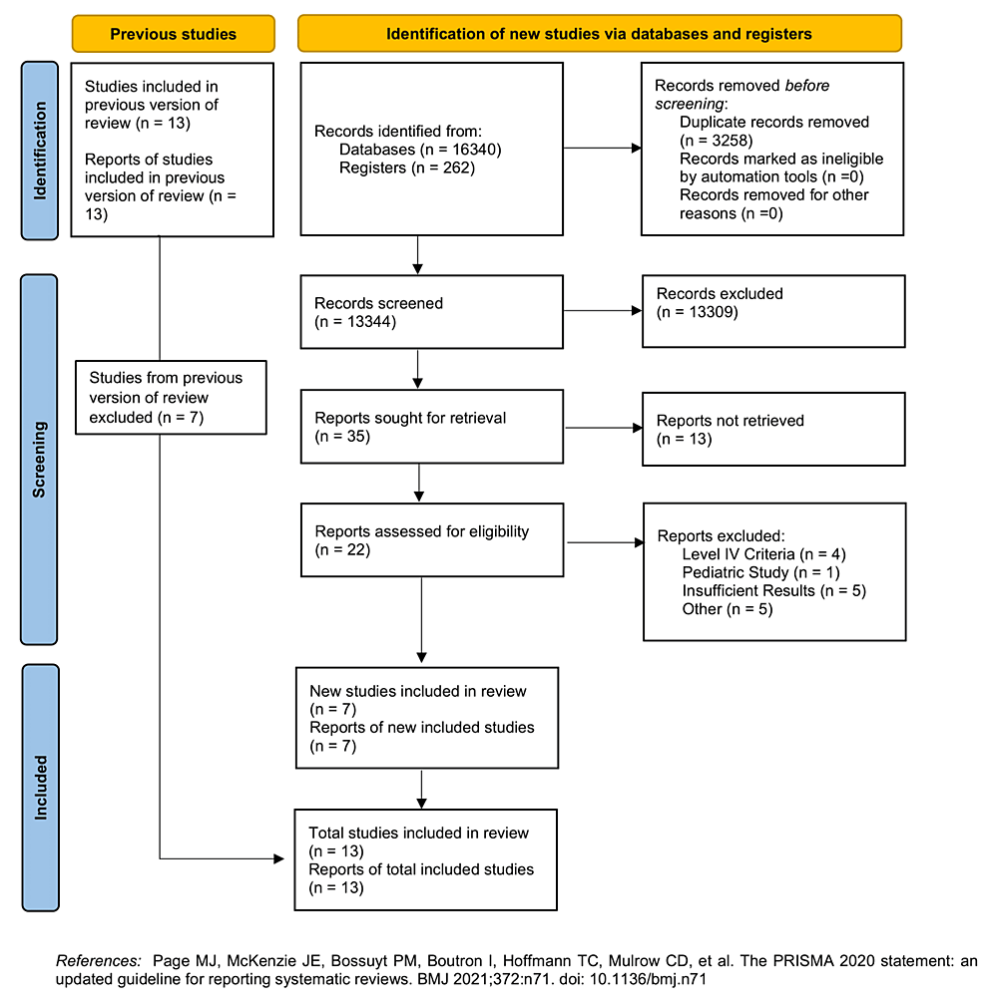
PRISMA (Preferred Reporting Items for Systematic Reviews and Meta-analyses) flow diagram of studies identified in the systematic review and meta-analysis. Source: [18].

A total of 16,602 appropriate studies were originally identified. After the elimination of duplicates and subsequent screening of records by title and abstract, 22 studies were chosen for the full-text assessment. Ultimately, 13 articles were used for meta-analysis, including five RCTs, three prospective cohort studies, and 5five retrospective cohort studies (Figure 1) [16,22-33]. The total number of patients across all included studies was 1,253 (576 in the intramedullary nail group and 677 in the LP group). The characteristics of all the included studies are summarized in Table 1.

**Table 1:** Characteristics of all included studies and patient demographics. RCT, randomized controlled trial; LPHP, low-profile locking proximal humerus; PHILOS, proximal humerus internal locking system

Study	Year	Nation	Study type	Diagnostic characteristics	Interventions	Sample size	Mean age (years)	Female (%)	Follow-up (months)	Rate of follow-up (%)
Zhu et al. [16]	2011	China	RCT	Displaced two-part fractures	Expert PHN (bend)	25	54.8	64	36	89
					LPHP/PHILOS plate	26	50.5	69.2	36	
Gradl et al. [22]	2009	Germany	Prospective	Displaced two-, three-, or four-part fractures	Targon PHN (straight)	76	63	68.4	12.8	74.8
					LPHP/PHILOS plate	76	63	68.4	12.8	
Konrad et al. [23]	2011	Germany/United Kingdom/Switzerland	Prospective	Displaced three-part fractures	Proximal humeral nail	58	64.8	81	12	84.4
					LPHP/PHILOS plate	158	65.4	74	12	
Urda et al. [24]	2012	Spain	Retrospective	Displaced two-part fractures	POLARUS nail (bend)	26	70.9	76	40.7	100
					PHILOS plate	15	71	80	40.7	
Tamimi et al. [25]	2015	Spain/Canada	Retrospective	Displaced two-, three-, or four-part fractures	Expert PHN (bend)	19	65.3	52.6	22.5	100
					PHILOS plate	44	65.3	65.9	25.9	
Gracitelli et al. [26]	2016	Brazil	RCT	Displaced two- or three-part fractures	Centronail (bend)	33	66.5	69	12	93.8
					PHILOS plate	32	66.4	76	12	
Ge et al. [27]	2017	China	Prospective	Displaced two- or three-part fractures	Intramedullary nail	79	76.89	69	24	92.9
					Locking plate	72	75.14	65	24	
Lee et al. [28]	2017	Korea	Retrospective	Displaced two-part fractures	POLARUS nail (bend)	38	59.7	68.4	24	100
					PHILOS plate	31	58.6	64.5	24	
Plath et al. [29]	2019	Germany	RCT	Displaced three-part or AO 11-B1 fractures	Locking blade nail (straight)	36	71.1	64	12	80.8
					PHILOS plate	32	77.1	68	12	
Setaro et al. [30]	2020	Italy	Retrospective	Displaced two- or three-part fractures	TRIGEN nail (bend)	53	64	Not reported	40.4	<100, “some patients lost to follow up”
					PHILOS plate	64	61.5	Not reported	48	
Boyer et al. [31]	2021	France	RCT	Displaced three- or four-part fractures	MultiLoc nail (straight)	43	74	57	66	83.5
					Surfix plate	42	77	53	66	
Wu et al. [32]	2021	China	RCT	Displaced two- or three-part fractures	MultiLoc nail (straight)	58	42.15	39.66	6	100
					PHILOS plate	57	43.29	35.09	6	
Lan et al. [33]	2022	China	Retrospective	Displaced three- or four-part fractures	Intramedullary nail	32	68.91	56.25	22	100
					LPHP plate	28	66.62	57.14	22	

Methodological Quality

The methodological quality of RCTs is detailed in Table 2 [20]. The quality of non-RCT studies was assessed using the MINORS appraisal score, averaging 18.5 +/- 2.07 (range 16-22) (Table 3), which showed moderate quality of the included studies [21].

**Table 2:** Risk-of-bias summary of all included RCTs. + represents yes; – represents no; ? represents not clear. RCT, randomized controlled trial

Zhu et al. (2011) [16]	Gracitelli et al. (2016) [26]	Plath et al. (2019) [29]	Boyer et al. (2021) [31]	Wu et al. (2021) [32]	
-	-	-	-	?	Random sequence generation (selection bias)
?	?	?	?	?	Allocation concealment (selection bias)
+	+	+	+	+	Binding of participants and personnel (performance bias)
+	-	-	+	+	Binding of outcome assessment (detection bias)
-	-	+	-	-	Incomplete outcome (attrition bias)
-	-	-	-	-	Selective reporting (reporting bias)
-	-	-	-	-	Other bias

**Table 3:** The MINORS appraisal scores for the included prospective and retrospective comparative studies. Methodological items are as follows: (1) a clearly stated aim; (2) inclusion of consecutive patients; (3) prospective collection of data; (4) endpoints appropriate to the aim of the study; (5) unbiased assessment of the study endpoint; (6) follow-up period appropriate to the aim of the study; (7) loss to follow-up, which is less than 5%; (8) prospective calculation of the study size; (9) an adequate control group; (10) contemporary groups; (11) baseline equivalence of groups; (12) adequate statistical analyses. The items are scored as *0* (not reported), *1* (reported but inadequate), or *2* (reported and adequate). The global ideal score for comparative studies is 24, all selected studies were greater than 15 indicating moderate to high quality of studies. MINORS, Methodological Index for Nonrandomized Studies

Name	Methodological Items												Total
	1	2	3	4	5	6	7	8	9	10	11	12	
Gradl et al. (2009) [22]	2	2	2	2	0	2	1	0	1	2	2	2	18
Konrad et al. (2011) [23]	2	2	2	2	0	1	0	2	1	2	2	2	18
Urda et al. (2012) [24]	2	2	2	2	0	2	2	1	1	2	2	2	20
Tamimi et al. (2015) [25]	2	2	2	2	0	1	0	1	1	2	2	2	17
Ge et al. (2017) [27]	2	2	2	2	0	1	1	2	1	2	2	2	19
Lee et al. (2017) [28]	2	2	2	2	2	2	2	1	1	2	2	2	22
Setaro et al. (2020) [30]	2	1	2	2	0	2	1	2	1	2	2	2	19
Lan et al. (2022) [33]	2	2	2	2	0	0	0	1	1	2	2	2	16

Functional Outcome Scores

**Figure 2:**
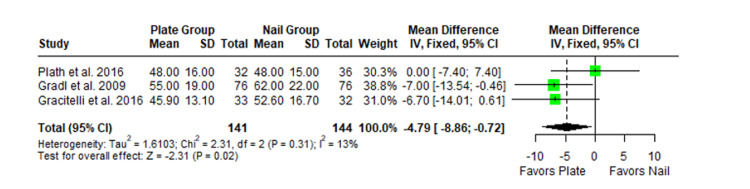
Forest plot showing the pooled mean difference between the locking plate and intramedullary nail group for Constant Murley scores at three months. Sources: [29,22,26]. SD, standard deviation; CI, confidence interval

**Figure 3:**
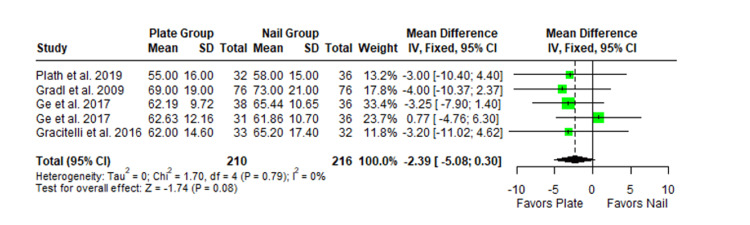
Forest plot showing the pooled mean difference between the locking plate and intramedullary nail groups for Constant Murley scores at six months. Sources: [29,22,27,26] SD, standard deviation; CI, confidence interval

**Figure 4:**
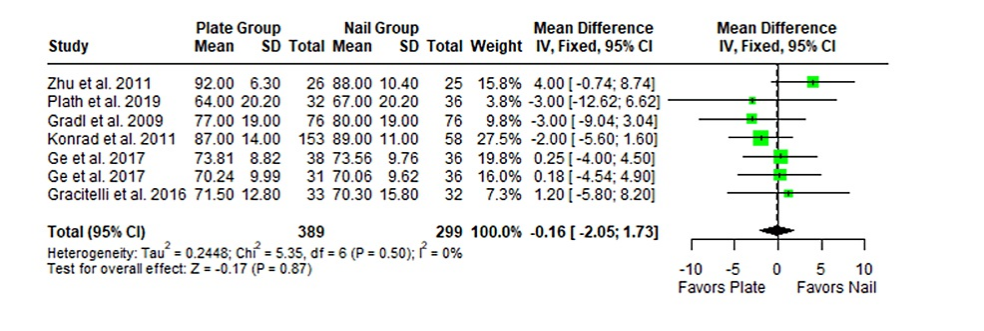
Forest plot showing the pooled mean difference between the locking plate and intramedullary nail groups for Constant Murley scores at 12 months only. Sources: [16,29,22,23,27,26]. SD, standard deviation; CI, confidence interval

**Figure 5:**
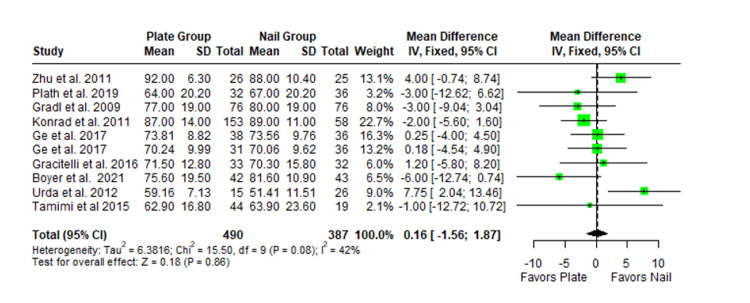
Forest plot showing the pooled mean difference between the locking plate and intramedullary nail groups for Constant Murley scores at 12 months and beyond. Sources: [16,29,22,23,27,26,31,24,25]. SD, standard deviation; CI, confidence interval

**Figure 6:**
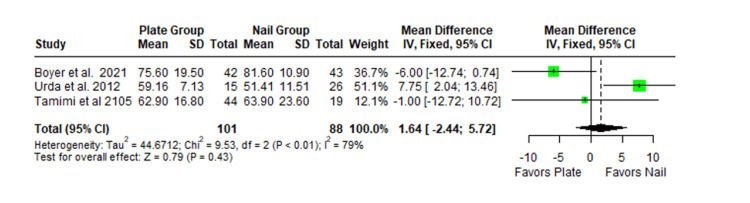
Forest plot showing the pooled mean difference between the locking plate and intramedullary nail groups for Constant Murley scores beyond 12 months only. Sources: [31,24,25]. SD, standard deviation; CI, confidence interval

**Figure 7:**
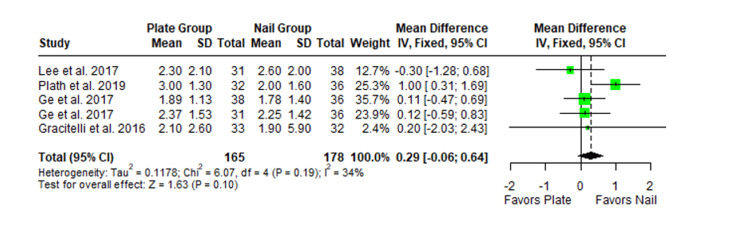
Forest plot showing the pooled mean difference between the locking plate and intramedullary nail groups for the visual analog scale (VAS) at six months. Sources: [28,29,27,26]. SD, standard deviation; CI, confidence interval

**Figure 8:**
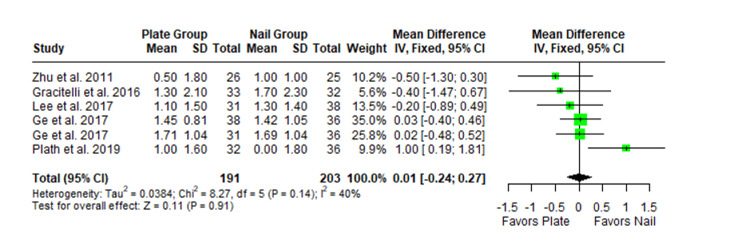
Forest plot showing the pooled mean difference between the locking plate and intramedullary nail groups for the visual analog scale (VAS) at 12 months. Sources: [16,26,28,27,29]. SD, standard deviation; CI, confidence interval

**Figure 9:**
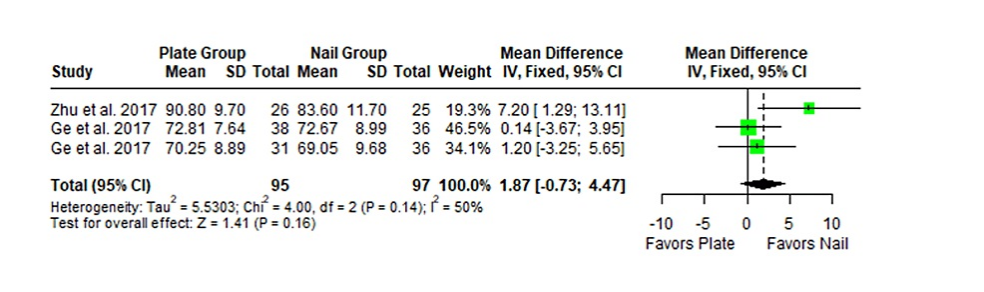
Forest plot showing the pooled mean difference between the locking plate and intramedullary nail groups for ASES score at 12 months only. Sources: [16,27]. SD, standard deviation; CI, confidence interval; ASES, American Shoulder and Elbow Surgeons

**Figure 10:**
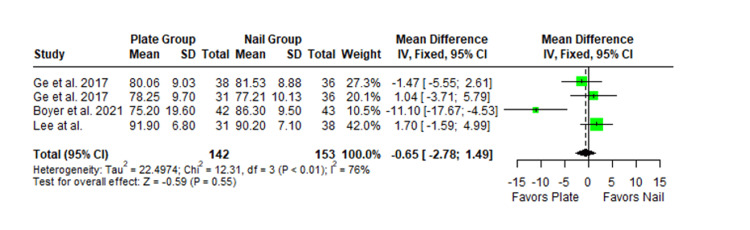
Forest plot showing the pooled mean difference between the locking plate and intramedullary nail groups for ASES score at 24-36 months. Sources: [27,31,28]. SD, standard deviation; CI, confidence interval; ASES, American Shoulder and Elbow Surgeons

**Figure 11:**
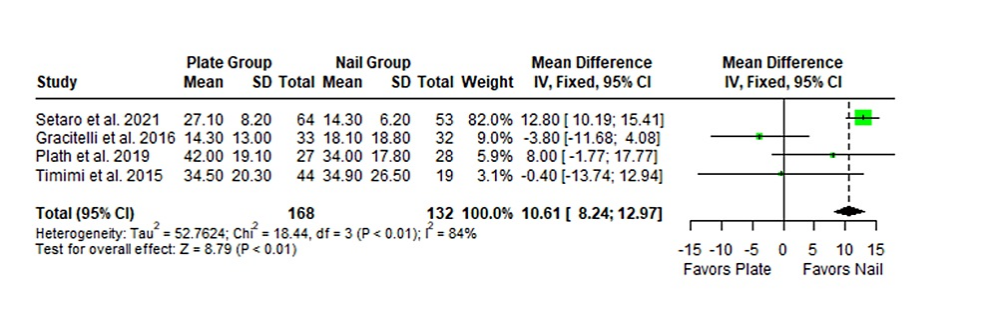
Forest plot showing the pooled mean difference between the locking plate and intramedullary nail groups for DASH score at 12 months. Sources: [30,26,29,25]. SD, standard deviation; CI, confidence interval; DASH, Disabilities of the Arm, Shoulder, and Hand

**Figure 12:**
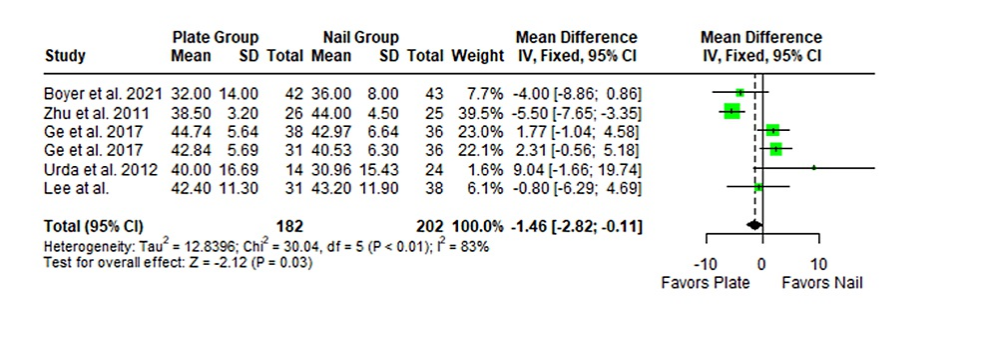
Forest plot showing the pooled mean difference between the locking plate and intramedullary nail groups for external rotation. Sources: [31,16,27,24,28]. SD, standard deviation; CI, confidence interval

**Figure 13:**
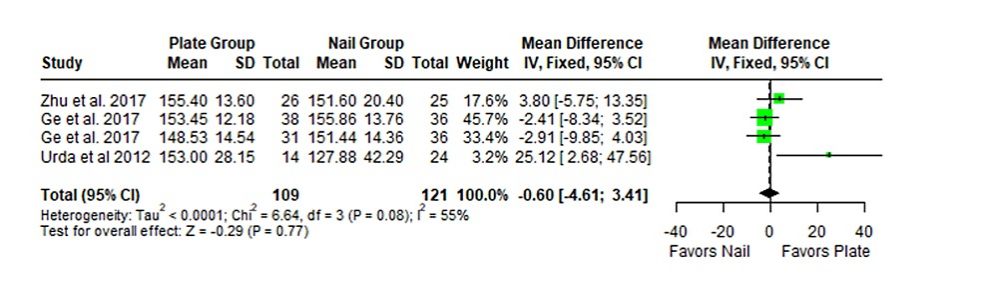
Forest plot showing the pooled mean difference between the locking plate and intramedullary nail groups for forward flexion. Sources: [16,27,24]. SD, standard deviation; CI, confidence interval

The Constant-Murley scores were significantly higher in the LP group compared to the intramedullary nail group at three months follow-up (MD -4.79; 95% CI -8.86 to -0.72; *P *= 0.02; I2 = 13%) (Figure 2). There was no significant difference observed at six months (MD -2.39; 95% CI -5.08 to 0.30; *P* = 0.08; I2 = 0%) (Figure 3). Finally, there was no significant difference observed at exactly 12 months (MD -0.16; 95% CI -2.05 to -1.73; *P *= 0.87; I2 = 0%) (Figure 4), greater than or equal to 12 months (MD 0.16; 95% CI -1.56 to 1.87; *P *= 0.86; I2 = 42%) (Figure 5) or beyond 12 months only (MD 1.64; 95% CI -2.44 to 5.72; *P*-value = 0.43; I2 = 79%) (Figure 6). There was also no significant difference observed for the VAS between the two groups at six months (MD 0.29; 95% CI -0.06 to 0.64; *P* = 0.10; I2 = 34%) (Figure 7) or at 12 months follow-up (MD 0.01; 95% CI -0.24 to 0.27; *P *= 0.91; I2 = 40%) (Figure 8). There was no significant difference seen between the two groups with regards to the ASES score at exactly 12 months (MD 1.87; 95% CI -0.73 to 4.47; *P *= 0.16; I2 = 50%) (Figure 9) or beyond 12 months (MD -0.65; 95% CI -2.78 to 1.49; *P *= 0.55; I2 = 76%) (Figure 10). The DASH score was significantly greater in the LP group as compared to the intramedullary nail group (MD 10.61; 95% CI 8.24 to 12.97; *P*<= 0.01; I2 = 84%) (Figure 11), and there was significantly greater external rotation observed in the plate group as compared to the intramedullary nail (MD -1.46; 95% CI -2.82 to -0.11; *P* = 0.03; I2 = 83%) (Figure 12). There was no significant difference between the two groups observed for forward flexion (MD -0.60; 95% CI -4.61 to 3.41; *P *= 0.77; I2 = 55%) (Figure 13). 

Complications

**Figure 14:**
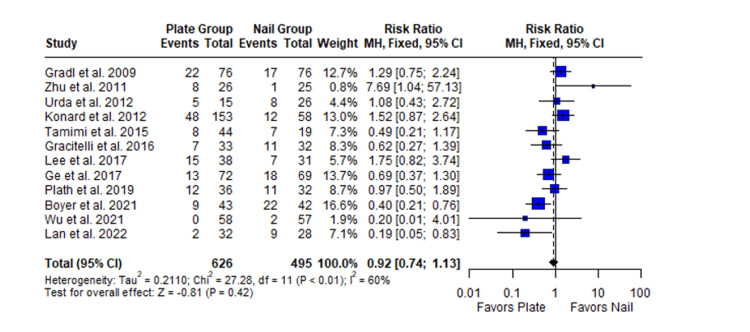
Forest plot showing the pooled mean difference between the locking plate and intramedullary nail groups for complications. Sources: [22,16,24,23,25,26,28,27,29,31,32,33]. SD, standard deviation; CI, confidence interval

The combined estimate of all complications revealed that there were no significant differences between the two groups (MD 0.92; 95% CI 0.74 to 1.13, *P*-value = 0.42, I2 = 60%) (Figure 14).

Discussion

PHFs are the third most common fracture in patients aged over 65 years old and the seventh most common fracture in adults [1]. With an aging population and an increase in the prevalence of PHFs, there should be a substantial effort to optimize both the non-operative and operative management of these patients [4]. Displaced PHFs are commonly treated with either intramedullary nails or LPs. However, superiority in terms of functional scores, pain scores, and total complication rates between the two methods has not yet been demonstrated [14]. A previous meta-analysis in 2018 showed no superior treatment between the two and only a greater shoulder external rotation and penetration rate with LPs [13]. This meta-analysis provides an update to the 2018 study with the addition of two more RCTs and more recent prospective and retrospective comparative studies.

Regarding functional scores, Sun et al. concluded equivalent functional scores for DASH (MD = 0.28; 95% CI −2.56 to 3.12; *P* = 0.27; I2 = 23%) and Constant-Murley scores (MD = −0.63; 95% CI −2.27 to 1.01;*P *= 0.22; I2 = 24%) [13]. In contrast, this study had a statistically significant DASH score favoring intramedullary nails at 12 months (MD = 10.61; 95% CI 8.24 to 12.97; *P* < 0.01; I2 = 84%) and Constant-Murley at three months favoring LPs (MD -4.79; 95% CI -8.86 to -0.72; *P* = 0.02; I2 = 13%). This could indicate less patient-perceived functional disabilities in the intramedullary nail group and better initial short-term functional outcomes in the LP group. One possible explanation for this difference could be attributed to the common approach for intramedullary nails involving deltoid splitting, as opposed to the deltopectoral approach used for LPs. This could lead to initial weakness of overhead motion, limiting activities of daily living. This is further exemplified by a significantly higher external rotation ROM in LP groups, potentially attributed to the rotator cuff violation in the intramedullary nail approach. The use of a straight versus proximal bend nail may have affected the rotator cuff and external rotation parameters. A straight nail starting point is more medial and is thought to spare the rotator cuff versus the more lateral starting point of the proximal bend nail. However, we are unable to elaborate on this further as only 10 out of the 13 studies reported the type of nail used, and only five studies included external rotation as an outcome measure. Furthermore, Constant-Murley scores at six months, 12 months, and beyond 12 months were not statistically significant, making the argument that long-term functional outcomes may be equivalent. Furthermore, ASES scores were not statistically significant, further suggesting similar functional outcomes between the two groups. Our findings are consistent with a recent 2023 systematic review and meta-analysis of surgical treatments of PHFs [34]. Hohmann et al. found that surgical treatment with LPs versus intramedullary nailing yielded no difference in long-term clinical outcomes and ROM. 

While VAS scores showed no statistically significant difference in this meta-analysis, Plath et al. demonstrated a significant difference in less pain at six months and 12 months, favoring intramedullary nailing [29]. Two included studies also favored intramedullary nailing at six months, although these findings did not achieve statistical significance [26,27]. This could indicate intramedullary nailing being better tolerated initially. Regarding the total complication rate, our meta-analysis yielded similar results to previous studies. Although various results, such as nonunion, infection, avascular necrosis, rotator cuff tear, and further surgery intervention yielded differences in the included studies, there was no significant difference in total complications between the two groups at 12 months follow-up. However, it is noted that not all studies were consistent with how complications were reported. Some studies reported the total number of complications, while others reported complications per patient. The inconsistent reporting may have skewed the data during the finalization of the total complications analysis. 

One limitation of our meta-analysis is a lack of stratification into two-, three-, or four-part fractures. A 2023 systematic review and meta-analysis by Lapner et al. showed a higher re-operation rate in LPs compared to hemiarthroplasty when stratified into three-part and four-part fractures [35]. For intramedullary nails versus LPs, the stratification of three-part and four-part fractures could yield different results and is a potential future study. Other limitations include breaking down the functional scores or complications into individual components, separation by age bracket, or high-energy versus low-energy trauma. These choices were made due to insufficient data in each more specific category. Furthermore, not every functional score and complication forest plots were stratified to consistent sub-groups such as three, six, or 12 months. This is due to differences in reporting of these scores at different time frames in the included studies.

## Conclusions

This systematic review and meta-analysis suggest a superior Constant-Murley score and functionality with locking plates for PHFs in the early postoperative period, as well as a greater external rotation ROM. However, long-term functional scores and complication rates were comparable between the two groups. This suggests that using intramedullary nails and LPs for PHFs are relatively equivalent, and there is currently no strong evidence for one over the other. There may be an early postoperative advantage in using LPs due to an earlier return to activities of daily living. However, these results could be due to differences in fracture characteristics, individual surgeon's skills, and patient's functional status. We suggest conducting additional RCTs comparing LPs and intramedullary nails to fully evaluate the differences.
